# What do Iranian physicians value most when choosing a specialty? Evidence from a discrete choice experiment

**DOI:** 10.1186/s12962-022-00358-z

**Published:** 2022-05-26

**Authors:** Yaser Sarikhani, Sulmaz Ghahramani, Sisira Edirippulige, Yoshikazu Fujisawa, Matthew Bambling, Peivand Bastani

**Affiliations:** 1 Research Center for Social Determinants of Health, Jahrom University of Medical Sciences, Jahrom, Iran; 2 Health Human Resources Research Center, School of Management and Information Sciences, Shiraz University of Medical Sciences, Almas Building, Alley 29, Qasrodasht Ave, Shiraz, Iran; 3 Health Policy Research Center, Institute of Health, Shiraz University of Medical Sciences, Shiraz, Iran; 4 Centre for Online Health, The University of Queensland, Brisbane, Australia; 5 School of Project Design, Miyagi University, Tokyo, Japan; 6 School of Clinical Medicine, Faculty of Medicine, The University of Queensland, Brisbane, Australia; 7 Oral Health Centre, School of Dentistry, Faculty of Health and Behavioural Sciences, The University of Queensland, Brisbane, Australia

**Keywords:** Discrete choice experiment, Specialty preferences, General physicians, Iran

## Abstract

**Background:**

Choosing a specialty by physicians is fundamentally linked to the performance of health systems and public health outcomes. Identifying the determinants of specialty selection is important to health policy for targeting resources and planning the development of services. This study examined preferences of Iranian physicians for medical specialty using a discrete choice experiment (DCE) method.

**Methods:**

In this study, the attributes of the DCE were determined using rigorous qualitative approach. Then we applied D-efficiency criteria to design the DCE and validated it at a pilot study. In the final survey, we recruited participants from six Iranian provinces and analyzed data using conditional logit model. We estimated willingness to pay (WTP) for non-monetary attributes.

**Results:**

The WTP analysis revealed that the most important non-monetary attributes in the selection of a specialty were job burnout, opportunity for procedural activities, and job prestige. The results imply that the attributes that were related to the quality of personal life was more important only for physicians who preferred to choose non-surgical specialties.

**Conclusions:**

The findings demonstrate that traditional gender patterns of specialty selection are changing and quality of personal life characteristics might be the most important factor when developing policies to recruit physicians into non-surgical specialties.

**Supplementary Information:**

The online version contains supplementary material available at 10.1186/s12962-022-00358-z.

## Background

The success of health systems is based on having a well-trained health workforce to ensure an acceptable level of service delivery [1]. Achieving the main goals of the health system, such as universal health coverage, depends on the quality, number, composition, and distribution of health workers [2]. Physicians are at the core of healthcare team and represent an essential resource for the provision of health services. Selection of specialty profoundly affects the composition of the physician workforce [3]. Several negative consequences are associated with choosing a specialty by physicians [4]. There are high numbers of physicians who prefer to enter specialized fields which may lead to a shortage of physicians in primary health care [5]. This results in over-specialization of medical services and greatly increases the cost of services and overall health expenditures [6]. Some specialties appear to be more popular than others, therefore a dominant preference toward particular fields may cause an unbalanced distribution of physicians in different specialties [4, 7]. Specialty imbalance affects the performance of the health system [8] and may limit equitable access to health services, particularly in remote and rural areas [9].

There are many factors involved in the selection of specialty by physicians [10]. Identifying these factors provides evidence for physician workforce planning that can predict growth trends and address shortfalls in various specialties [6, 9, 11]. Studies demonstrate that personal determinants [12–14], financial considerations [15–17], educational aspects [18–20], and job features [8, 18, 21] are among the influential factors in the selection of specialty. Career characteristics are among the main determinants of specialty selection [21]. There are several ways to explore the impact of career-related determinants on the selection of a specialty. Investigating the preferences of health staff regarding job characteristics is a well-known approach [22]. In this regard, discrete choice experiment (DCE) has been widely used in health-related studies [23]. Although applying DCE to investigate physician preference for a career specialty is a new approach, its use is increasing due to the quality of information elicited [24]. DCE has also been proposed as a preferential method to inform health workforce policy in developing countries [25].

Iran, as a developing country, faces many challenges regarding the physician workforce [26]. In addition to the shortage of physicians [27], the country is also suffering from the problem of an unbalanced distribution of physicians in various specialty fields and different geographical areas [26, 28–30]. Because of the importance of providing precise evidence for physician workforce policy, a national survey was undertaken to examine the preferences of Iranian physicians for the selection of specialty applying DCE.

## Methods

In this study, data collection was carried out from January to July 2020 in Iran. We used DCE as a stated-preference method [31] to elicit preferences of Iranian physicians for the selection of a specialty applying three methodological frameworks for the design [32], implementation [33], and validation [34] of DCE surveys. The flowchart of this study is presented in Additional file 1: Appendix S1.

### Identification of attributes and levels

Preferences for a career are dependent on the specific characteristics of the career and for the purposes of DCE these are referred to as attributes. Moreover, a range of aspects called levels usually describes each attribute. In this study, preference attributes and levels were developed using the framework developed by Helter and Boehler [33]. This framework consists of three steps, including (A) raw data collection, (B) data reduction, (C) removing inappropriate attributes, and wording of attributes.

(A) Raw data Gathering: In this step, a review study to determine the main factors associated with choosing a specialty as a career was undertaken. A systematic search and qualitative synthesis of the data in the form of a scoping review was undertaken using the approach proposed by Arksey and O’Malley [35]. The framework proposed for scoping review consists of five steps, including 1—determining the research question, 2—finding related studies, 3—selecting relevant studies, 4—extracting and charting the data, as well as 5—collating, summarizing and reporting the findings. A qualitative content analysis was used to summarize data in the review phase. The main themes that emerged from the scoping review were used to develop a topic guide for the qualitative study. The findings of the review study are published in a separate report [36]. In the second stage, 41 in-depth interviews were conducted with medical students (N: 12), junior doctors (N: 11), and medical residents (N: 18) to identify the most important determinants of specialty selection in Iran. All participants were recruited using a purposeful sampling method at Shiraz University of Medical Sciences. Data collection was based on open-ended face to face interviews and transcripts were analyzed using thematic content analysis. In both the review and qualitative studies, to ensure the quality of the results, we used the Guba and Lincoln criteria, including credibility, dependability, transferability, and confirmability [37]. We tried to improve the credibility of the results, through prolonged engagement with the data, using member check and a peer debriefing approach. The dependability of the study was ensured by detailed documentation of the research process as well as through external audit of the study process by four colleagues. We tried to confirm transferability by providing precise descriptions of the method. Finally, to improve confirmability, two qualitative researchers were asked to verify the accuracy of the study process and data synthesis.
(B) Data reduction: In the second stage, informed by the findings of the previous phase, a joint meeting was undertaken with Iranian experts in medical education and health economics to select career-related factors that are suitable for developing the DCE. As a result, a comprehensive list of job characteristics was prepared.
(C) Removing redundant attributes and wording of attributes: Achieving an appropriate number of attributes and levels is a concern noted in previous DCE studies [38]. Two sessions of nominal group technique (NGT) were conducted to finalize the attributes and levels of the DCE using the methodology proposed by Potter et al. [39]. The protocol proposed by Potter et al. consists of five steps, including (1) introduction and explanation of the purpose, (2) silent generation of ideas, (3) sharing ideas, (4) group discussion, and (5) voting and ranking. Twelve participants from the qualitative study attended NGTs. In the first session, participants were provided with the list of job characteristics obtained from the previous stage and to rate the list (from 1 to 5) based on four criteria of importance, reasonability, least possible collinearity, and the possibility of a trade-off between the levels [9, 40]. The group selected eight characteristics with an average score above half of the total score (i.e., 30) as attributes of the DCE. In the second NGT session, the participants were asked to provide several lists of levels for each attribute independently. Participants then rated each list of levels from 1 (least priority) to 5 (highest priority) based on the four criteria used for the rating of the attributes. Accordingly, the lists of levels with an average score above half of the total score (i.e., 30) were selected. Finally, the list of levels was determined so that two attributes were assigned four levels, four attributes were assigned three levels, and two attributes were assigned two levels. In this session, the group checked the wording of the attributes and levels and amended them where it was necessary. Moreover, the group was asked to determine the effect of each attribute on the selection of a specialty as a priori expectations that was used for the validation of the questionnaires.

### Specifying the preference elicitation approach

In this study, we used a DCE because this method has been suggested as a useful technique for preference elicitation in health-related studies [32]. A DCE involves some choice tasks, and each choice task includes two or more choices that are the result of the combination of attribute levels.

### Task construction and experimental design

I this study we used a fractional factorial design (FRFD) to develop choice tasks. Based on the number of attributes and levels in this study, a full design would lead to 5184 (4^2^ × 3^4^ × 2^2^) possible choices and accordingly 5184 × 5183/2 possible choice tasks. Because it was impossible to use a full design, a FRFD was applied considering D-efficiency criteria [25]. We used SAS software (version 9.4; SAS Institute, Inc., Cary, North Carolina) for experimental design, and allowed to construct 36 choice tacks randomly divided into four blocks [41]. In the construction of choice tasks, a paired forced-choice design was used without involving an opt-out choice to maximize the possibility of trade-offs and obtain more data about the importance of the attributes. Finally, a generic labeling (specialty A and B) was applied to avoid the effect of prejudice in favor of a particular specialty [31].

The process and the results of the experimental design are available in a separate report published as the study protocol [42].

### Questionnaire design and pilot study

Base on the number of blocks, four questionnaires were developed in Persian. The first part of all questionnaires consisted of some questions on demographic characteristics, a description of the DCE, a guide to the questionnaire, and definitions for all attributes. The second part involved nine main choice tasks and tasks added for validity and reliability assessment. An example of choice tasks in English is presented in the protocol of the study [42].

To evaluate the validity and reliability of the instrument, a pilot study among physicians from Fars province of Iran was conducted. Those who were preparing for the medical residency exam were included in the pilot study. Based on the rule of the thumb that suggest a minimum of 20 samples per independent variable in logistic regression models, a sample size of 240 junior doctors was targeted (30 participants for each attribute as an independent variable) [43]. A multistage proportionate cluster random sampling was used to select participants. The list of the physicians in the province was obtained from the Medical Council of the Islamic Republic of Iran (IRIMC). Sampling was performed in different settings, including personal offices, hospitals, polyclinics and medical universities. All participants completed questionnaires in person. The quality of the questionnaires was assessed using the framework proposed by Janssen et al. [34]. The priori expectations was evaluated against the model coefficients to determine the theoretical validity of the design [44]. To estimate coefficients, a conditional logit model (CLM) using STATA software (version 15.1; StataCorp, LP, College Station, TX) was used [25]. Two tests to evaluate the reliability of the instrument were undertaken. Firstly, we presented the same choice task to the participants twice to evaluate response consistency [34]. Secondly, a fixed-choice task was added to all versions of the questionnaires to evaluate version consistency [34]. To investigate the validity of the responses, we assessed task non-attendance considering those who selected all options from one side of the choice tasks [34]. Finally, a dominant choice task was added to examine the rationality of the responses [34]. To improve the validity of the results, those who had task non-attendance and those who did not give a logical answer to the dominant choice (choosing dominated scenario in the dominant choice task) were not included in the final analysis. Since the pilot study was conducted merely to evaluate the validity of the experiment and to confirm the priori expectations, the data gathered in the pilot study were not included in the final survey.

### Data collection

After confirming the validity and reliability of the questionnaires, the main study was conducted in the six provinces of the country, including Tehran in the north, Fars in the south, Khorasan-Razavi in the northeast, Kerman in the southeast, Kermanshah in the west, and Khuzestan in the southwest. These provinces were selected from different geographic regions with various socio-economic conditions to ensure maximum diversity. The population of general physicians in the six provinces was more than 51% (N: 48,964) of the total population of general physicians in the country at the time of the study.

In this research, the study population (defined as physician) included all junior doctors and practicing physicians with active clinical practice license who were seeking a residency in a specialty. The sample size of the study was based on the Sudman population statistic of a maximum of 100 participants per 10,000 target population [45]. Because of the number of the target population in this study (N: 48,964), it was sufficient to recruit 480 physicians. However, to improve the power of estimations, a sample size of 720 was targeted. A multistage random cluster sampling method was used to aid recruiting participants. Firstly, a complete list of physicians in the six provinces was collated based on the data provided by IRIMC. Secondly, in each province the geographic regions were categorized based on their population. In the third stage, each category was divided into two clusters based on gender. Finally, a proportional random sampling in each gender category was undertaken. Willingness to attend to a specialty course and preparing to take the medical residency exam and willingness to participate in the study were considered as inclusion criteria. Based on the prepared list, respondents were selected from different settings, including personal offices, hospitals, polyclinics and medical universities.

We made Initial phone calls with physicians randomly based on the lists for each cluster and determined those who were eligible and agreed to participate in the study. Then all participants filled out the questionnaires in a face-to-face setting.

### Statistical analysis

In this study we used logit model to estimate parameters. The logit model has been suggested in DCE studies due to the flexibility of the approach [25]. Using the logit model, the probability of choosing specialty *i* is specified as:

1 $${\text{p}}_i=\frac{\text{exp}\left(V_i\right)}{{\sum\nolimits_{j=1}^n} {\text{exp}}\left(V_j\right)}$$

In this study, we estimated the parameters using a CLM because this approach allows a better insight into the observed variations, which can provide better information for policymakers [25]. We also considered mixed-logit model (MLM) as an option for data analysis. Preliminary analysis of MLM was performed incorporating two variables of gender and type of specialty (surgical or non-surgical) that were identified in the initial qualitative studies as two important determinants of specialty selection. However, the results of MLM showed that the effect of gender and type of specialty was not significant. Accordingly, only the results of CLM are presented in this report. The results of MLM are reported in Additional file 2: Appendix S2.

In this study, we also estimated the monetary value of the attributes of a specialty using willingness to pay (WTP). WTP indicates how much monthly salary a physician is willing to sacrifice to have an improvement in a non-monetary aspect of the specialty. We estimated WTP as:

$${\text{WT}}{{\text{P}}_{lxi}} = - \frac{{{{\partial U} \mathord{\left/ {\vphantom {{\partial U} {{\partial _{lxi}}}}} \right. \kern-\nulldelimiterspace} {{\partial _{lxi}}}}}}{{{{\partial U} \mathord{\left/ {\vphantom {{\partial U} {{\partial _{income}}}}} \right. \kern-\nulldelimiterspace} {{\partial _{income}}}}}} = - \frac{{{\beta _{lxi}}}}{{{\beta _{income}}}}$$

where *lxi* is an attribute level.

In the estimation of WTP, we considered the income attribute as a continuous variable. The delta approach was used to estimate a 95% confidence interval for WTP values [46]. In this study, we also simulated potential selection of specialty. The simulation shows how the probability of choosing a given specialty changes as levels of an attribute are improved. Although many variables are known to be involved in choosing a specialty, based on the findings of qualitative and review phases of the study, it was hypothesized that the two main subgroups may respond differently to the policies. Therefore, we also performed the simulation for two subgroups of gender and type of specialty preferred. In order to avoid information overload, the results of simulation model are presented in Additional file 3: Appendix S3.

To estimate coefficients, we used STATA software (version 15.1; StataCorp, LP, College Station, TX). The full protocol of this study and the results of the pilot phase have been published in another report [42].

## Results

In the pilot study, data was gathered from 240 physicians with a mean age of 32.01 ± SD = 4.70. More than 53% (N: 129) of the participants were male. Two hundred and twenty-five (n = 225) physicians (93.75%) gave a logical answer to the dominant choice and the rate of task non-attendance was 3.75% (N: 9), which seems to be acceptable for a DCE. In this regard, those with task non-attendance and those who gave an irrational response to the dominant choice (choosing dominated scenario in the dominant choice task) were excluded from the final analysis (N: 17, 7.08%). Findings indicated that 221 physicians (87.08%) chose repeat-choice tasks the same way, indicating that the measurement had acceptable reliability during the survey. Moreover, the results of the chi-squared test showed that there was no significant difference between blocks in the selection of choices of the fixed-choice tasks (P: 0.639), indicating that the design has version consistency. Results of the logit model showed that all attributes were significant in explaining selection of a specialty. We also found that the signs of all estimated coefficients were compatible with the priori expectations, revealing that the design has theoretical validity. Table 1 shows the results of the pilot study model.

**Table 1:** Results of the pilot study model This table is taken from the authors’ previous report on the study protocol [42]

Attributes and levels	β (95% CI)^a^	A priori expectations
Expected monthly income		Generally, a positive preference is assumed for higher income
200 million (Iranian Rial) IRR^b^	–	
300 million IRR	0.89 (0.70–1.07)*	
500 million IRR	1.40 (1.18–1.62)*	
700 million IRR	1.80 (1.57–2.04)**	
Opportunity for procedural activity		Generally, a positive preference is assumed for greater opportunity for procedural activity
Great	–	
Ordinary	− 0.27 (− 0.49 to − 0.05)**	
Rare	− 0.65 (− 0.85 to − 0.46)*	
Work-family compatibility		Generally, a positive preference is assumed for higher work-family compatibility
High	–	
Relative	− 0.48 (− 0.63 to − 0.33)*	
Low	− 0.76 (− 0.96 to − 0.57)*	
Opportunity for academic careers		Generally, a positive preference is assumed for greater academic and research opportunity
Great	–	
Ordinary	− 0.60 (− 0.78 to − 0.41)*	
Undesirable	− 0.73 (− 0.94 to − 0.53)*	
Occupational prestige		Generally, a positive preference is assumed for higher job prestige
Excellent	–	
Ordinary	− 0.82 (− 0.95 to − 0.70)*	
Job burnout		Generally, a negative preference is assumed for higher job burnout
High	–	
Medium	0.79 (0.57–1.01)*	
Low	0.96 (0.76–1.17)*	
Very low	1.31 (1.08–1.54)*	
Emergency or on-call schedule		Generally, a negative preference is assumed for more on-call cases
Often	–	
Sometimes	0.21 (0.01–0.41)*	
Seldom	0.51 (0.32–0.70)*	
Scope of practice		Generally, a positive preference is assumed for broader scope of practice
Broad	–	
Narrow	− 0.30 (− 0.43 to − 0.17)*	
Constant	0.11 (0.03–0.023)*	

Number of observations: 4014, LR chi^2^(16) = 227.49, Prob > chi^2^ = 0.000, Pseudo R^2^ = 0.384

*Significant at P < 0.0001

**Significant at P < 0.05

^a^Model coefficients and corresponding confidence interval

^b^The exchange rate of Iranian rial at the time of the study was 150,000 rials per US dollar

In the final study, 720 physicians from the six provinces were recruited. The mean age of participants was 31.83 ± SD = 4.38 with 53% (N: 382) of participants being male. Table 2 indicates the characteristics of physicians participated in the DCE. After excluding those who had task non-attendance and those with an irrational response to the dominant choice (N: 38, 5.27%), data from 682 physicians was included in the final analysis.

**Table 2:** Characteristics of the DCE participants

Characteristics	Total^a^	Males^a^	Females^a^
Age			
26–35	575 (79.9)	278 (72.8)	297 (87.8)
36–45	145 (21.1)	104 (27.2)	41 (12.2)
Marital status			
Married	448 (62.2)	243 (63.6)	205 (60.7)
Single	272 (33.7)	139 (36.4)	133 (39.3)
Living location			
Population < 100,000	82 (11.4)	43 (11.2)	39 (11.5)
Population > 100,000	124 (17.2)	65 (17.0)	59 (17.5)
Provincial centers	514 (71.4)	274 (71.8)	240 (71.0)
Practice setting			
Salaried	341 (47.4)	180 (47.1)	161 (47.6)
Self-employed	238 (33.1)	121 (31.7)	117 (34.6)
No job	141 (19.5)	81 (21.2)	60 (17.8)
Preferred specialty			
Surgical	421 (58.5)	250 (65.4)	171 (50.6)
Non-surgical	299 (41.5)	132 (34.6)	167(49.4)

^a^Number and percent

The results of the logit model presented in Table 3 revealed that with the improvement of all attribute levels from the baseline levels, except for an ordinary opportunity for academic careers, the preferences for the selection of a specialty have increased significantly (P < 0.0001). The highest preferences, compared to the base levels were related to income levels of more than 500 million Iranian Rials (IRR), lower job burnout, and higher job prestige.

**Table 3:** Preferences for specialty attributes

Attribute levels^a^	*β* (SE)^b^	OR (95% CI)^c^	P-value
Monthly income of 300,000,000 IRR	0.73 (0.05)	2.08 (1.86–2.33)	< 0.0001
Monthly income of 500,000,000 IRR	1.49 (0.06)	4.44 (3.88–5.07)	< 0.0001
Monthly income of 700,000,000 IRR	1.73 (0.07)	5.65 (4.92–6.50)	< 0.0001
Opportunity for procedural activity (ordinary)	0.42 (0.05)	1.53 (1.37–1.70)	< 0.0001
Opportunity for procedural activity (great)	0.71 (0.06)	2.03 (1.80–2.29)	< 0.0001
Work-family compatibility (relative)	0.35 (0.04)	1.14 (1.01–1.23)	< 0.0001
Work-family compatibility (high)	0.41 (0.05)	1.42 (1.29–1.56)	< 0.0001
Opportunity for academic careers (ordinary)	− 0.01 (0.05)	1.01 (0.90–1.14)	0.780
Opportunity for academic careers (great)	0.71 (0.06)	2.05 (1.81–2.32)	< 0.0001
Job prestige (excellent)	0.75 (0.03)	2.13 (1.97–2.30)	< 0.0001
Job burnout (medium)	0.78 (0.06)	2.19 (1.92–2.50)	< 0.0001
Job burnout (low)	0.94 (0.06)	2.56 (2.25–2.90)	< 0.0001
Job burnout (very low)	1.21 (0.06)	3.38 (2.95–3.87)	< 0.0001
Emergency or on-call schedule (sometimes)	0.35 (0.06)	1.42 (1.26–1.60)	< 0.0001
Emergency or on-call schedule (seldom)	0.45 (0.05)	1.57 (1.40–1.75)	< 0.0001
Scope of practice (broad)	0.32 (0.03)	1.37 (1.28–1.48)	< 0.0001
Constant	0.13 (0.03)	–	< 0.0001

Number of observations = 12,276; LR chi^2^(15) = 2417.68; Prob > chi^2^ = 0.0000; Pseudo R^2^ = 0.2841

^a^Baseline levels: monthly income (200,000,000 IRR), opportunity for procedural activity (undesirable), work-family compatibility (low), opportunity for academic careers (undesirable), job prestige (ordinary), job burnout (high), emergency or on-call schedule (often), scope of practice (narrow)

^b^Model coefficient and corresponding standard error

^c^Odds ratio and corresponding confidence interval

In order to present evidence in a more policy-focused manner, the WTP analysis (Table 4) showed that Iranian physicians were willing to give up 354 million IRR monthly income to select specialties with very low job burnout instead of specialties with high job burnout. This amount was equivalent to 259 and 203 million IRR for low and medium job burnout, respectively. The results also revealed that WTP for ordinary and great opportunity for procedural activity in place of the baseline level were 151 and 278 million IRR, respectively. On average, Iranian physicians were willing to reduce their monthly income by 222 million IRR if they could select a specialty with excellent prestige. Although the coefficient of WTP for the ordinary opportunity for academic careers was not significant, the participants have an average willingness to give up 210 million IRR monthly income to select specialties with a great opportunity for academic careers. Iranian physicians were willing to forgo 121 and 115 million IRR monthly income for specialties with higher work-family compatibility and less emergency or on-call schedule. Finally, they were willing to sacrifice the lowest amount of monthly income (0.7 million IRR) for specialties with a broader scope of practice instead of specialties with a limited scope of practice. In other words, to compensate for the worst level of specificity selection attributes, physicians should be paid more for high job burnout, undesirable opportunity for procedural activity, ordinary job prestige, undesirable opportunity for academic careers, low work-family compatibility, higher amount of emergency or on-call schedule, and narrow scope of practice, respectively.

**Table 4:** Willingness to pay for an improvement in the attribute levels

Attributes^a^	Willingness to pay^b^	95% confidence interval	
		Lower level	Upper level
Opportunity for procedural activity (ordinary)	1.5127	1.1821	1.8433
Opportunity for procedural activity (great)	2.7821	2.3815	3.1827
Work-family compatibility (relative)	1.0322	0.8595	1.2874
Work-family compatibility (high)	1.2162	0.9103	1.5220
Opportunity for academic careers (ordinary)	− 0.0755^c^	− 0.4272	0.2762
Opportunity for academic careers (great)	2.1061	1.7222	2.4900
Job prestige (excellent)	2.2211	1.9733	2.4690
Job burnout (medium)	2.0356	1.6589	2.4123
Job burnout (low)	2.5900	2.2130	2.9669
Job burnout (very low)	3.5456	3.1566	3.9345
Emergency or on-call schedule (sometimes)	0.7074	0.3614	1.0534
Emergency or on-call schedule (seldom)	1.1582	0.8222	1.4942
Scope of practice (broad)	0.7965	0.5674	1.0256

Number of observations = 12,276; LR chi^2^(15) = 2304.89; Prob > chi^2^ = 0.0000; Pseudo R^2^ = 0.2709

^a^Baseline levels: Monthly income (200,000,000 IRR), opportunity for procedural activity (undesirable), work-family compatibility (low), opportunity for academic careers (undesirable), job prestige (ordinary), job burnout (high), emergency or on-call schedule (often), scope of practice (narrow)

^b^Estimated based on the expected monthly income divided by 100,000,000 IRR and considered as a continuous variable

^c^Not significant at 0.05

## Discussion

This study examined the preferences of Iranian physicians for selecting a specialty. In this regard we used DCE, which provides accurate evidence for policy making [25]. However, for policy implications, marginal estimates are usually more important in an economic analysis [46]. Therefore, two important questions were evident. First, how the probability of choosing specialty changes when a specialty attribute alters? Second, which policy is more effective in the selection of specialty by physicians? Accordingly, we adopted two approaches to answer these questions. Firstly, we estimated WTP for all non-monetary attributes to explore how much monthly salary were physicians willing to sacrifice for a higher level of an attribute. Secondly, we investigated the changes in the probability of selecting a specialty resulting from improvement in the levels of specialty attributes. Although many variables were involved in the selection of specialty, based on the findings of the review and qualitative phases of the study, an assumption was made that the two main subgroups might react differently to various policies. As a result, we repeated the second approach in terms of gender and type of preferred specialty.

The CLM revealed that the highest preferences relative to the baseline levels were related to the higher income levels, lower job burnout, higher job prestige, greater opportunity for procedural activities, and greater opportunity for academic careers, respectively. However, fewer emergency or on-call schedules, higher work-family compatibility, and a broader scope of practice were valued less over the baseline levels. Many DCE studies have shown the effect of income-related preferences on the selection of a job or a specialty [6, 47–50]. However, the effect of intangible factors such as prestige and burnout have not been directly explored in DCEs. Only a DCE by Cleland et al. indicated that clinical or academic reputation is one of the determinants of career selection by UK doctors [51]. It has been reported that job prestige is somewhat dependent on the income level of a career [52]. Therefore, the financial aspect, which is widely discussed in similar studies, can indirectly explain job prestige. Also, it may be assumed that job burnout and work-family compatibility can be determined by factors such as working hours [6, 50, 52], control on hours [6, 52, 53], working environment [47, 54], and working condition [51, 55] that have been discussed in several DCE surveys. Although the effect of the scope of practice on the selection of specialty is widely reported by several related articles [56–58], no DCE investigated this factor.

The results of the present study are supported by the existing research [56, 59–63], and suggest that while intangible characteristics may not be directly examined [52], their importance should not be overlooked by researchers and policymakers. Since attributes of DCEs are determined on specific contextual conditions, the potential differences must be taken into account in the interpretation and application of the results.

The analysis of willingness to pay indicated that the highest amounts of monthly income that Iranian physicians were willing to sacrifice were related to low job burnout, a greater opportunity of procedural activity, and job prestige. The lowest amounts of WTP were related to the higher work-family compatibility, fewer emergency or on-call schedules, and broader scope of practice. These findings are confirmed by other DCEs which reported a significant willingness to pay for more procedural activities [6, 52], less on-call programs [47, 64], higher job prestige [51], and the higher possibility of academic activities [6, 49] when physicians are going to choose a career or a specialty. However, the results of this study regarding the willingness of Iranian physicians to pay for specialties with higher work-family compatibility and less emergency schedules were unexpected as participants in the early qualitative phase emphasized these two factors as important. In this regard, it is expected that investigating the monetary value of attributes of this DCE in terms of various demographic and specialty subgroups may result in different findings. Generally, findings of WTP analysis imply that the most important non-pecuniary attributes in the selection of specialty by Iranian physicians were job burnout, opportunity for procedural activities, job prestige, and opportunity for academic careers, respectively. These factors may be important when developing policies related to recruiting physicians in different specialties or encouraging them to practice as primary care physicians.

### Policy implications

The results imply that three characteristics, including monthly income, opportunity for procedural activities, and job burnout had the highest weight in the analysis of preferences for choosing a specialty by Iranian physicians. The findings suggest that these attributes should be given more attention in formulating national reform plans aimed at changing the composition of the specialist physician workforce.

In the related literature, gender has been described as a determining factor in selecting specialty [5, 61, 65]. In the current study, female physicians, compared to males, attributed more weight to the most attributes except scope of practice and emergency or on-call schedule. As several studies have shown that factors such as income, prestige, and procedural activities are more important to male physicians, results of this study may indicate that the pattern of choosing a specialty among women is changing. These findings are consistent with a study by Scanlan et al. which showed that gender norms of career selection are changing [66]. National policies could be developed to address undesirable gender trends in the selection of specialty courses.

Many review studies have reported that there are significant differences in the preferences of physicians for surgical and non-surgical specialties [5, 61, 65, 67]. The current study indicated that choosing a non-surgical specialty was mainly associated with three characteristics, including work-family compatibility, job burnout, and emergency or on-call schedule. These findings are in line with several related studies [5, 60, 65, 67, 68]. In recent years, particular high-paying specialties have attracted more attention in Iran, which may lead to a shortage of physicians in other fields such as primary health care. Therefore, the results of this study may assist in formulating policies aimed at the balanced distribution of physicians between surgical and non-surgical specialties.

### Strengths and limitations of the study

Attributes and levels of this DCE were determined based on rigorous qualitative studies to ensure the quality of the design and validity of the results. Moreover, the study was carried out in different geographical regions of the country using a multistage random cluster sampling approach to increase the generalizability of the results. The main limitation of this study is that preferences of specialty selection only explored in the general population of physicians and two main subgroups. However, the analysis of preferences according to various specialty disciplines may have different results. Finally, it should be noted that this study was conducted during the coronavirus disease-2019 (COVID-19) pandemic and this may have influenced the preferences of the participants.

## Conclusions

Selection of a specialty career by physicians is a complex process that has many factors involved. In this study, a DCE was used to elicit preferences of Iranian physicians for the selection of specialty. In general, the highest preferences of Iranian physicians were related to higher income, lower job burnout, higher job prestige, and greater opportunity for procedural activities, respectively. The marginal WTP analysis revealed that the most important non-monetary attributes in the selection of a specialty were job burnout, opportunity for procedural activities, and job prestige, respectively. Moreover, the results imply that usual gender patterns in the specialty selection might be changing. Characteristics related to the quality of personal life were more important for physicians who preferred to choose non-surgical specialties. Although this study provided some evidence for physician workforce policy, analysis of preferences according to different specialty fields remains an area for further investigation.

## Supplementary Information

**Additional file 1. **Flowchart of the study.

**Additional file 2. **The results of CLM.

**Additional file 3. **The results of simulation model.

## Data Availability

The datasets used and analyzed during the current study are available from the corresponding author on reasonable request.
