# Supplementary figures and images for: What do Iranian physicians value most when choosing a specialty? Evidence from a discrete choice experiment
Source: Cost Eff Resour Alloc. 2022 May 26;20:23. doi: 10.1186/s12962-022-00358-z (PMC9134140; doi:10.1186/s12962-022-00358-z)

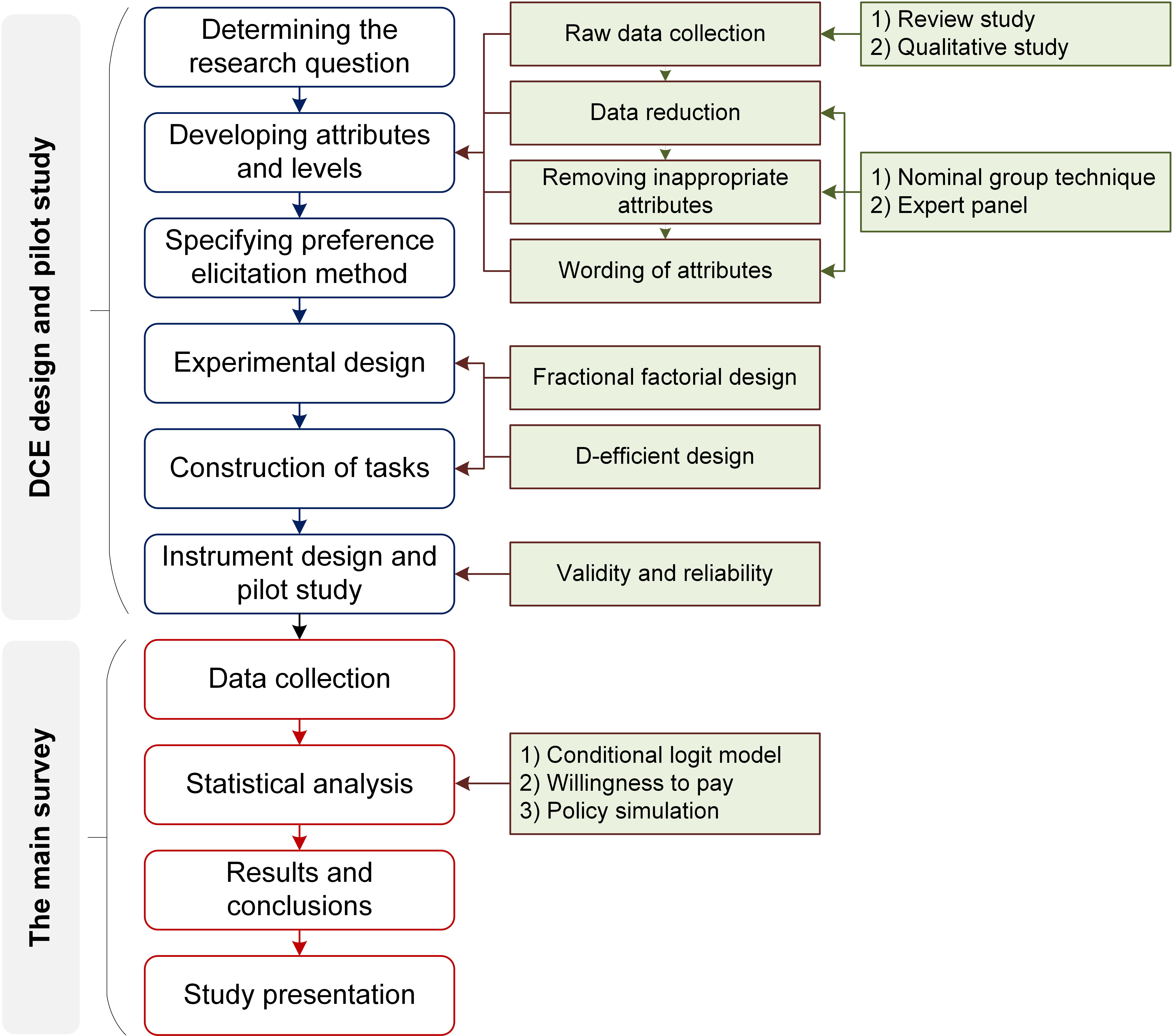

Supplement: Supplementary file 1 — Additional file 1. Flowchart of the study. [file 12962_2022_358_MOESM1_ESM.jpg]
