# Supplementary material for: What do Iranian physicians value most when choosing a specialty? Evidence from a discrete choice experiment
Source: Cost Eff Resour Alloc. 2022 May 26;20:23. doi: 10.1186/s12962-022-00358-z (PMC9134140; doi:10.1186/s12962-022-00358-z)
Supplement: Supplementary file 2 — Additional file 2. The results of CLM. [file 12962_2022_358_MOESM2_ESM.docx]

| **Syntax codes of mixed-logit model for SAS software** |
| --- |
| proc bchoice data=data seed=1 nmc=30000 thin=2  nthreads=2 DIC diag=(AutoCorr ESS MCSE);  class Gender (ref='1') Prefered_Spec(ref='1') att1(ref='1') att2(ref='1') att3(ref='1') att4(ref='1') att5(ref='1') att6(ref='1') att7(ref='1') att8(ref='1')id paircode;  model ch = att1 att2 att3 att4 att5 att6 att7 att8 Age/ choiceset=(id paircode);  random Gender Prefered_Spec / subject=id monitor=(1 to 2)  type=un;  run; |

| **Model Information** | |
| --- | --- |
| **Data Set** | WORK.DATA |
| **Response Variable** | ch |
| **Type of Model** | Logit |
| **Fixed Effects Included** | Yes |
| **Random Effects Included** | Yes |
| **Sampling Algorithm** | Gamerman Metropolis |
| **Burn-In Size** | 500 |
| **Simulation Size** | 30000 |
| **Thinning** | 2 |
| **Random Number Seed** | 1 |
| **Number of Threads** | 2 |

| **Class Level Information** | | |
| --- | --- | --- |
| **Class** | **Levels** | **Values** |
| **att1** | 4 | 1 2 3 4 |
| **att2** | 3 | 1 2 3 |
| **att3** | 3 | 1 2 3 |
| **att4** | 3 | 1 2 3 |
| **att5** | 2 | 1 2 |
| **att6** | 4 | 1 2 3 4 |
| **att7** | 3 | 1 2 3 |
| **att8** | 2 | 1 2 |
| **Gender** | 2 | 1 2 |
| **Prefered_Spec** | 2 | 1 2 |

| **Choice Sets Summary** | | | | |
| --- | --- | --- | --- | --- |
| **Pattern** | **Choice Sets** | **Total Alternatives** | **Chosen Alternatives** | **Not Chosen** |
| **1** | 6138 | 2 | 1 | 1 |

| **Priors for Covariance Parameters of Random Effects** | | | |
| --- | --- | --- | --- |
| **Parameter** | **Prior Distribution** | **DF** | **Scale** |
| **RECov** | Inverse Wishart | 5 | 5 |

Results of the mixed-logit model

| **Posterior Summaries and Intervals** | | | | | |
| --- | --- | --- | --- | --- | --- |
| **Parameter** | **N** | **Mean** | **Standard Deviation** | **95% HPD Interval** | |
| **att1 2** | 15000 | 0.7752 | 0.0561 | 0.6593 | 0.8780 |
| **att1 3** | 15000 | 1.4995 | 0.0694 | 1.3616 | 1.6317 |
| **att1 4** | 15000 | 1.7679 | 0.0715 | 1.6306 | 1.9096 |
| **att2 2** | 15000 | -0.3168 | 0.0648 | -0.4382 | -0.1846 |
| **att2 3** | 15000 | -0.7670 | 0.0582 | -0.8810 | -0.6537 |
| **att3 2** | 15000 | -0.7705 | 0.0592 | -0.8902 | -0.6588 |
| **att3 3** | 15000 | -0.3876 | 0.0464 | -0.4779 | -0.2994 |
| **att4 2** | 15000 | -0.7368 | 0.0572 | -0.8487 | -0.6267 |
| **att4 3** | 15000 | -0.7449 | 0.0626 | -0.8643 | -0.6202 |
| **att5 2** | 15000 | -0.7935 | 0.0383 | -0.8715 | -0.7192 |
| **att6 2** | 15000 | 0.8125 | 0.0674 | 0.6857 | 0.9466 |
| **att6 3** | 15000 | 0.8988 | 0.0626 | 0.7767 | 1.0229 |
| **att6 4** | 15000 | 1.2470 | 0.0695 | 1.1134 | 1.3825 |
| **att7 2** | 15000 | 0.3478 | 0.0616 | 0.2254 | 0.4662 |
| **att7 3** | 15000 | 0.4794 | 0.0562 | 0.3730 | 0.5894 |
| **att8 2** | 15000 | -0.3281 | 0.0387 | -0.4058 | -0.2531 |
| **Age** | 15000 | -7.9365 | 6.0388 | -19.9563 | 0.5834 |
| **RECov Gender 2, Gender 2** | 15000 | 2.7683 | 4.7306 | 0.2472 | 9.0637 |
| **RECov Prefered_Spec 2, Gender 2** | 15000 | 0.4781 | 2.7185 | -3.1571 | 5.4315 |
| **RECov Prefered_Spec 2, Prefered_Spec 2** | 15000 | 2.2524 | 2.6772 | 0.2559 | 6.6822 |

The results of mixed-logit model showed that the effect of gender and type of specialty was not significant.
