# Supplementary material for: What do Iranian physicians value most when choosing a specialty? Evidence from a discrete choice experiment
Source: Cost Eff Resour Alloc. 2022 May 26;20:23. doi: 10.1186/s12962-022-00358-z (PMC9134140; doi:10.1186/s12962-022-00358-z)
Supplement: Supplementary file 3 — Additional file 3. The results of simulation model. [file 12962_2022_358_MOESM3_ESM.docx]

**Policy simulation**

In this study, we simulated potential selection of specialty to provide useful information for policymakers. The simulation shows how the probability of choosing a given specialty changes as levels of an attribute are improved. In this regard, the reference category of all attributes is considered as a baseline specialty. Then we estimated the probability of specialty selection due to the changes in the baseline attribute levels. Although many variables are known to be involved in choosing a specialty, based on the findings of qualitative and review phases of the study, it was hypothesized that the two main subgroups may respond differently to the policies. Therefore, we also performed the simulation for two subgroups of gender and type of specialty preferred. The probability of choosing specialty *i* rather than *j* is estimated as:

$P_{i}=\frac{e^{\beta^{'}x_{i}}}{\sum e^{\beta^{'}x_{i}}}$

Where *x* is a vector of attribute coefficients.

Findings from policy simulation in the whole sample (the following table) showed that the greatest changes in the probability of choosing a specialty was related to the changes in the levels of monthly income, opportunity for procedural activity, and job burnout, respectively. Simulation in the gender subgroup indicated that female physicians, compared to male physicians, had greater changes in the probability of choosing a specialty in all attributes except the emergency or on-call schedule and scope of practice. Finally, the simulation revealed that those physicians who preferred to select non-surgical specialties, compared to physicians who preferred surgical specialties had greater changes in the probability of choosing a specialty due to the changes in the levels of work-family compatibility, job burnout, and emergency or on-call schedule.

Changes in probability of choosing a specialty by change in each level from the baseline scenario

| **Attributes^*^** | **Total Sample^†^** | **Gender** | | **Preferred Field** | |
| --- | --- | --- | --- | --- | --- |
|  |  | **Male** | **Female** | **Surgical** | **Non-Surgical** |
| Monthly income of 300,000,000 IRI | **0.157**  (0.006) | **0.161**  (0.008) | **0.171**  (0.010) | **0.019**  (0.008) | **0.132**  (0.010) |
| Monthly income of 500,000,000 IRI | **0.443**  (0.015) | **0.452**  (0.019) | **0.472**  (0.024) | **0.522**  (0.018) | **0.379**  (0.028) |
| Monthly income of 700,000,000 IRI | **0.660**  (0.017) | **0.671**  (0.022) | **0.698**  (0.026) | **0.747**  (0.019) | **0.582**  (0.036) |
| Opportunity for procedural activity (Ordinary) | **0.235**  (0.024) | **0.281**  (0.034) | **0.233**  (0.038) | **0.223**  (0.036) | **0.359**  (0.040) |
| Opportunity for procedural activity (Great) | **0.415**  (0.024) | **0.436**  (0.033) | **0.455**  (0.038) | **0.638**  (0.027) | **0.161**  (0.043) |
| Work-family compatibility (Relative) | **0.147**  (0.027) | **0.238**  (0.036) | **0.018**  (0.034) | **0.062**  (0.034) | **0.121**  (0.044) |
| Work-family compatibility (High) | **0.190**  (0.023) | **0.290**  (0.038) | **0.327**  (0.034) | **0.026^‡^**  (0.036) | **0.432**  (0.032) |
| Opportunity for academic careers (Ordinary) | **-0.011^‡^**  (0.028) | **-0.207^‡^**  (0.038) | **0.227**  (0.043) | **-0.002^‡^**  (0.039) | **0.028^‡^**  (0.048) |
| Opportunity for academic careers (Great) | **0.322**  (0.027) | **0.178**  (0.039) | **0.519**  (0.037) | **0.471**  (0.035) | **0.167**  (0.045) |
| Job prestige (Excellent) | **0.338**  (0.016) | **0.320**  (0.023) | **0.403**  (0.024) | **0.430**  (0.024) | **0.294**  (0.026) |
| Job burnout (Medium) | **0.312**  (0.028) | **0.420**  (0.034) | **0.160**  (0.045) | **0.163**  (0.043) | **0.418**  (0.044) |
| Job burnout (Low) | **0.389**  (0.026) | **0.435**  (0.036) | **0.343**  (0.043) | **0.264**  (0.040) | **0.692**  (0.029) |
| Job burnout (Very low) | **0.510**  (0.024) | **0.481**  (0.034) | **0.555**  (0.034) | **0.309**  (0.039) | **0.761**  (0.025) |
| Emergency or on-call schedule (Sometimes) | **0.111**  (0.027) | **0.119**  (0.038) | **0.103**  (0.042) | **-0.073^‡^**  (0.039) | **0.364**  (0.034) |
| Emergency or on-call schedule (Seldom) | **0.181**  (0.026) | **0.340**  (0.33) | **0.008^‡^**  (0.041) | **0.096**  (0.039) | **0.384**  (0.039) |
| Scope of practice (Broad) | **0.125**  (0.017) | **0.215**  (0.023) | **0.005^‡^**  (0.027) | **0.176**  (0.024) | **0.159**  (0.030) |
| Number of observations | 12,276 | 6,516 | 5,760 | 7,182 | 5,094 |
| LR chi^2^ | 2304.8 | 1259.4 | 1340.7 | 1798.5 | 1125.8 |
| Prob>chi^2^ | 0.0000 | 0.0000 | 0.0000 | 0.0000 | 0.0000 |
| Pseudo R^2^ | 0.2709 | 0.2789 | 0.3358 | 0.3613 | 0.3189 |
| * Baseline scenario: Monthly income (200,000,000 IRR), Opportunity for procedural activity (Undesirable), Work-family compatibility (Low), Opportunity for academic careers (Undesirable), Job prestige (Ordinary), Job burnout (High), Emergency or on-call schedule (Often), Scope of practice (Narrow)  † Percent of change from baseline level and corresponding standard error  ‡ Not significant at 0.05 | | | | | |
